# Palsy of Both the Tibial Nerve and Common Peroneal Nerve Caused by a Ganglion Cyst in the Popliteal Area

**DOI:** 10.3390/medicina60060876

**Published:** 2024-05-27

**Authors:** Sang-Heon Lee, Sung-Hwan Kim, Ho-Sung Kim, Hyun-Uk Lee

**Affiliations:** Department of Orthopaedic Surgery, Soonchunhyang University Hospital Bucheon, 170, Jomaru-ro, Wonmi-gu, Bucheon-si 14584, Gyeonggi-do, Republic of Korea; worldking70@naver.com (S.-H.L.); shk9528@naver.com (S.-H.K.); nine4141@naver.com (H.-S.K.)

**Keywords:** tibial nerve, common peroneal nerve, ganglion cyst, ultrasound

## Abstract

A ganglion cyst is a benign mass consisting of high-viscosity mucinous fluid. It can originate from the sheath of a tendon, peripheral nerve, or joint capsule. Compressive neuropathy caused by a ganglion cyst is rarely reported, with the majority of documented cases involving peroneal nerve palsy. To date, cases demonstrating both peroneal and tibial nerve palsies resulting from a ganglion cyst forming on a branch of the sciatic nerve have not been reported. In this paper, we present the case of a 74-year-old man visiting an outpatient clinic complaining of left-sided foot drop and sensory loss in the lower extremity, a lack of strength in his left leg, and a decrease in sensation in the leg for the past month without any history of trauma. Ankle dorsiflexion and great toe extension strength on the left side were Grade I. Ankle plantar flexion and great toe flexion were Grade II. We suspected peroneal and tibial nerve palsy and performed a screening ultrasound, which is inexpensive and rapid. In the operative field, several cysts were discovered, originating at the site where the sciatic nerve splits into peroneal and tibial nerves. After successful surgical decompression and a series of rehabilitation procedures, the patient’s neurological symptoms improved. There was no recurrence.

## 1. Introduction

A ganglion cyst is a benign mass consisting of high-viscosity mucinous fluid. The mucinous fluid is surrounded by a collagenous fibrous capsule. Ganglion cysts can originate from the sheath of a tendon, peripheral nerve, or joint capsule [1]. Their peak incidence has been observed in individuals in their 40s. It is rare in children [2]. Although there is no definitive consensus on the risk and predisposing factors for ganglion cyst formation, it is known that they are more common in women than in men, particularly affecting those aged between 20 and 50 years. Ganglion cysts are especially prevalent among athletes, such as gymnasts, who experience repetitive stress on their joints [3]. Ganglion cysts are considered the most common tumor of the upper extremity. They occur frequently in the lower extremity and other parts of the body. Despite their high incidence, they rarely cause peripheral nerve compression [4]. Compression neuropathies caused by ganglion cysts are much less common. Most frequently, ganglion cysts involve the common peroneal nerve (CPN) and, less commonly, the ulnar and radial nerves [5,6]. In the upper extremities, ganglia have been reported to compress the ulnar nerve in Guyon’s canal, in the cubital tunnel, or the median nerve at the carpal tunnel [6]. The involvement of the lower extremities is significantly less common than the upper extremities. The compression of the peroneal nerve at the level of the knee and proximal tibiofibular joint has been described infrequently.

The etiology of peroneal intraneural ganglion cysts remains unidentified. Recently, the evidence has predominantly supported the synovial theory, which posits that intraneural ganglion cysts can develop due to a retrograde flow of synovial fluid from the knee joint. This flow occurs through a capsular defect into the epineurium of an articular branch of the peroneal nerve and subsequently into the CPN [7,8].

Sultan described a neuropathy of the peroneal nerve in 1921 [9]. Since then, few cases have been described in the literature. However, the reported cases have consistently involved individuals presenting with foot drop due to deep peroneal nerve palsy. To date, there have been no documented cases in which ganglion cysts forming on branches of the sciatic nerve, which bifurcates into the peroneal and tibial nerves, result in both peroneal and tibial nerve palsies simultaneously [1,7,10,11]. Although palsies of the peroneal nerve caused by ganglion cysts are uncommon, early diagnosis and prompt treatment are important if they do occur [12]. In this paper, we present the case of a 74-year-old man visiting the outpatient clinic complaining of left-sided foot drop, loss of ankle plantarflexion and toe flexion, and sensory loss in the lower extremity, indicating both tibial and peroneal nerve palsies.

## 2. Case Presentation

### 2.1. Preoperative Evaluation

**Figure 1:**
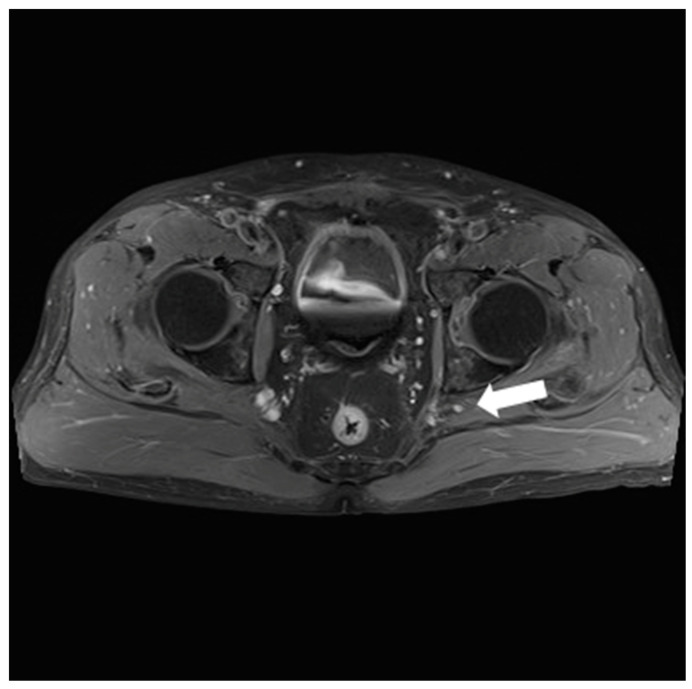
Hip T2-weighted sagittal magnetic resonance imaging (MRI) revealing a cyst measuring 0.5 cm × 0.5 cm next to the sciatic nerve (arrow).

A 74-year-old man visited a neurologist, complaining of a lack of strength in his left leg and a decrease in sensation in the leg for the past month. The neurology department performed an electromyography (EMG) test. The result indicated a sciatic nerve injury at the hip level. The nerve conduction study (NCS) results revealed a decreased sensory nerve action potential (SNAP) amplitude in the left tibial motor NCS and an inadequate formation of the F-wave waveform in left peroneal and tibial nerves. Subsequently, an evaluation of the sciatic nerve was conducted. The hip T2-weighted sagittal magnetic resonance imaging (MRI) revealed a cyst measuring 0.5 cm × 0.5 cm next to the sciatic nerve (Figure 1). The patient was referred to our orthopedic surgery department for the surgical treatment of the small hip cyst.

**Figure 2:**
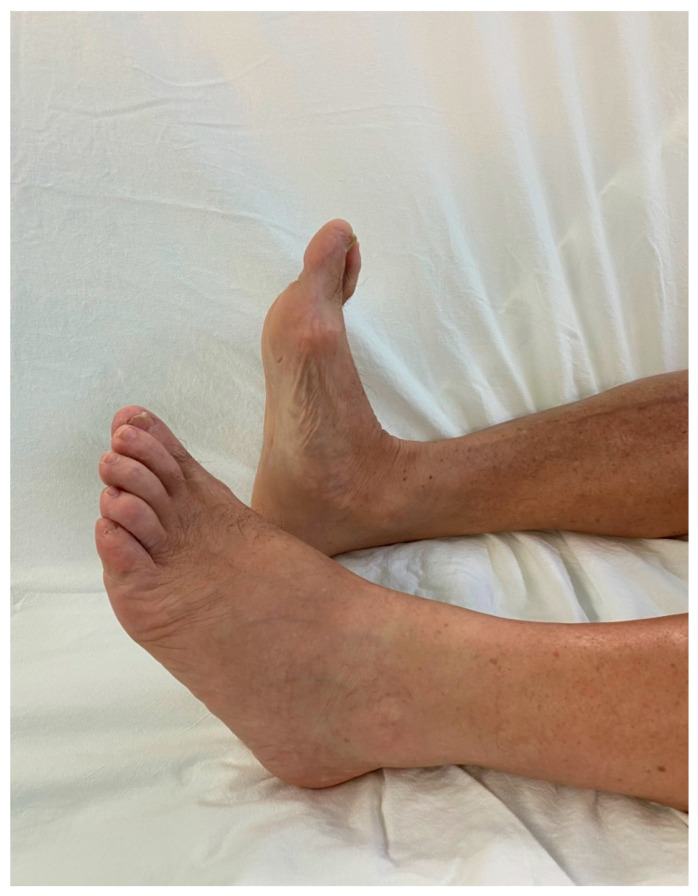
Grade I ankle dorsiflexion and great toe extension.

**Figure 3:**
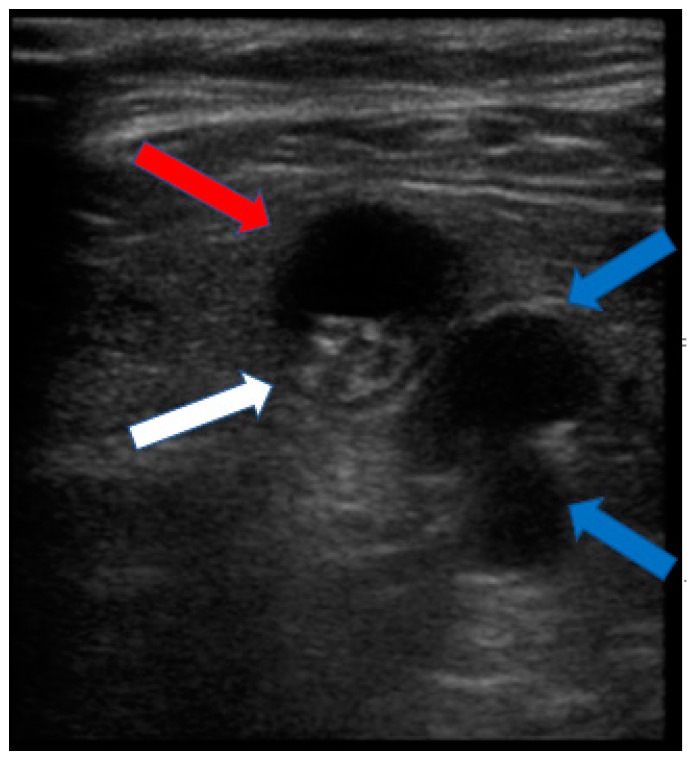
Ultrasound showing several cysts compressing the peroneal nerve around the distal femur level (red arrow: ganglion cyst; white arrow: compressed peroneal nerve; blue arrow: popliteal artery and vein).

**Figure 4:**
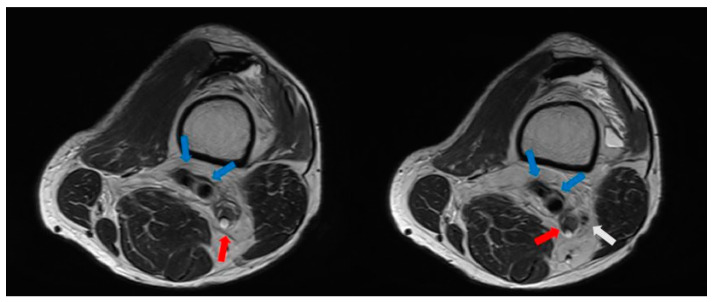
A preoperative knee T2-weighted axial MRI revealing ganglion cysts compressing the sciatic nerve at the level of bifurcation to the tibial nerve and the common peroneal nerve (red arrow: ganglion cyst; white arrow: common peroneal nerve; blue arrow: popliteal artery and vein).

**Figure 5:**
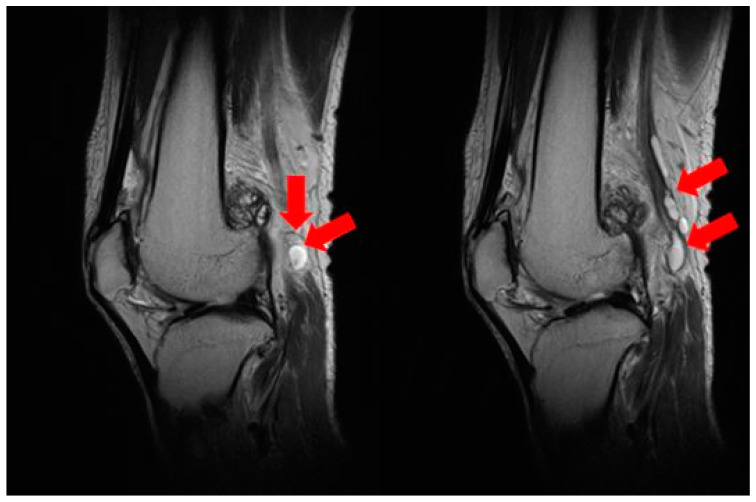
A preoperative knee T2-weighted sagittal MRI showing multiple ganglion cysts (red arrow).

**Figure 6:**
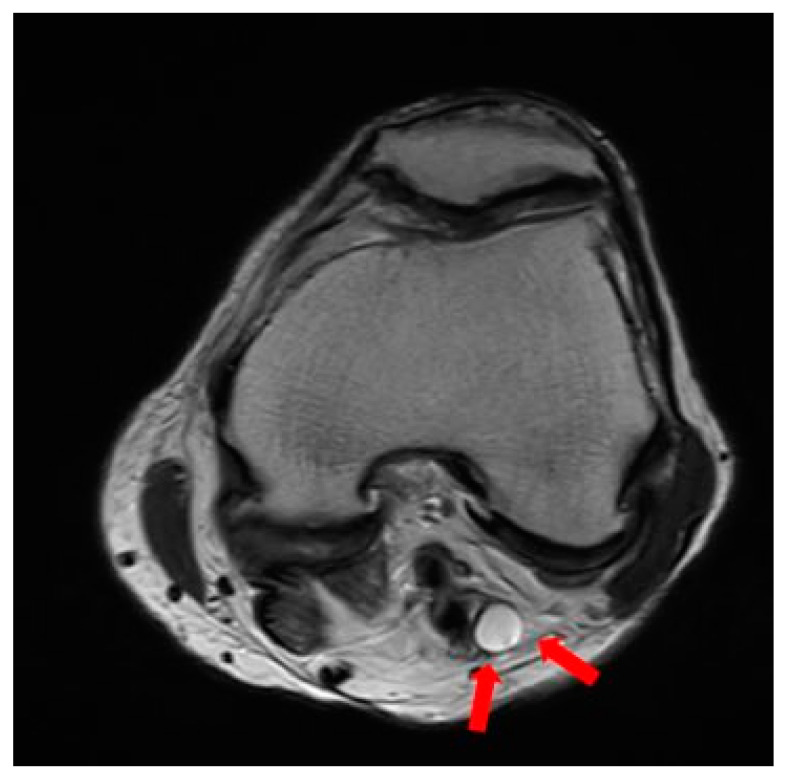
A preoperative knee T2-weighted axial MRI revealing ganglion cysts compressing the base of the tibial nerve (red arrow).

The patient had no history of any other trauma. When we conducted a physical examination, there were no abrasions or lacerations in the left lower extremity. Ankle dorsiflexion and great toe extension strength on the left side were Grade I (presence of muscle contraction but no joint movement, Figure 2). Ankle plantar flexion and great toe flexion were Grade II (movement was possible without the influence of gravity). Passive extension and flexion of the ankle and foot were intact without limitations in motion. There was a sensory deficit of 1/10 in the entire lower foot area below the ankle level. Considering the physical examination results, we suspected that the EMG result might be incorrect because his symptoms were only present below the ankle level. When various physical examinations that could detect spinal diseases were performed, they all yielded negative results. When we performed the Tinel test around the fibula head area, it was positive. Combining results of the physical examination, the patient exhibited symptoms of tibial nerve palsy, with ankle plantarflexion and great toe flexion (graded as II) decreased. Additionally, ankle dorsiflexion and great toe extension graded as I showed marked decreases along with a positive Tinel sign indicating peroneal palsy symptoms. Thus, a screening ultrasonography (USG) was performed by the attending orthopedic surgeon in the outpatient setting. As a result, it was found that several cysts were compressing the peroneal nerve around the distal femur level (Figure 3). These cysts exhibited characteristics such as being well-delineated and smoothly bordered and having homogeneous features, raising the possibility of synovial cysts and ganglion cysts. However, to differentiate from other lesions including seromas and to accurately diagnose and determine the extent of the lesion, an MRI was performed [13,14]. When we performed the T2-weighted sagittal MRI of the knee, we were able to identify multiple ganglion cysts compressing nerves at the sciatic nerve bifurcation where it branches into the tibial nerve and peroneal nerve (Figure 4 and Figure 5). Additionally, we confirmed the presence of a ganglion cyst compressing the nerve at the base of the tibial nerve, which branches off from the sciatic nerve (Figure 6). Methods like steroid injections or cyst aspiration may be considered; however, given the risks of nerve damage and high recurrence rates, and considering the severe neurological symptoms of the patient, surgery was decided upon [11,15,16,17].

### 2.2. Surgical Procedure

**Figure 7:**
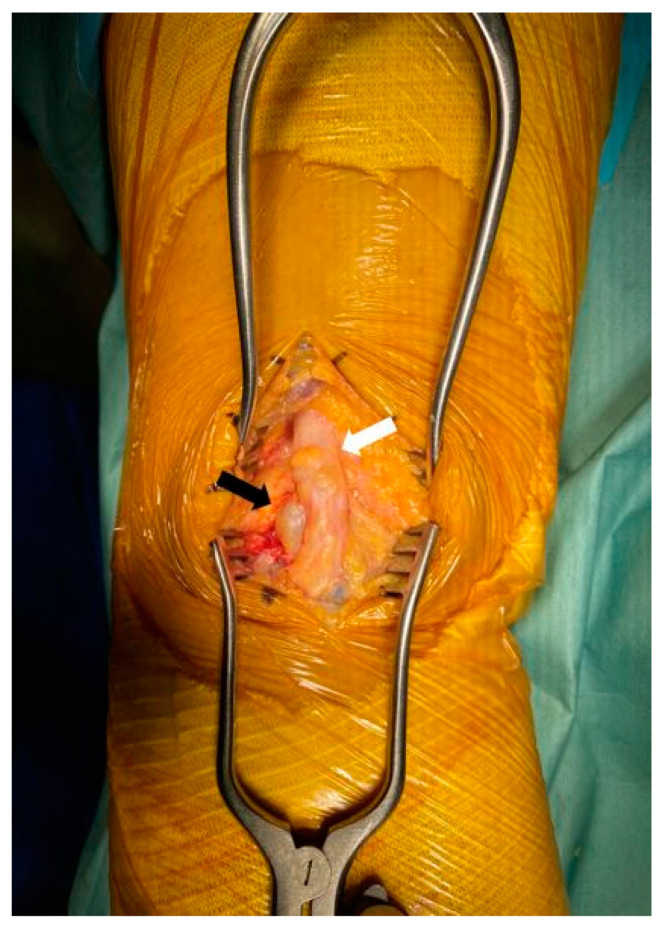
In operative field, multiple lobulated, 7 cm sized ganglion cysts were wrapped around the base of the tibia nerve (black arrow: ganglion cyst; white arrow: tibial nerve).

**Figure 8:**
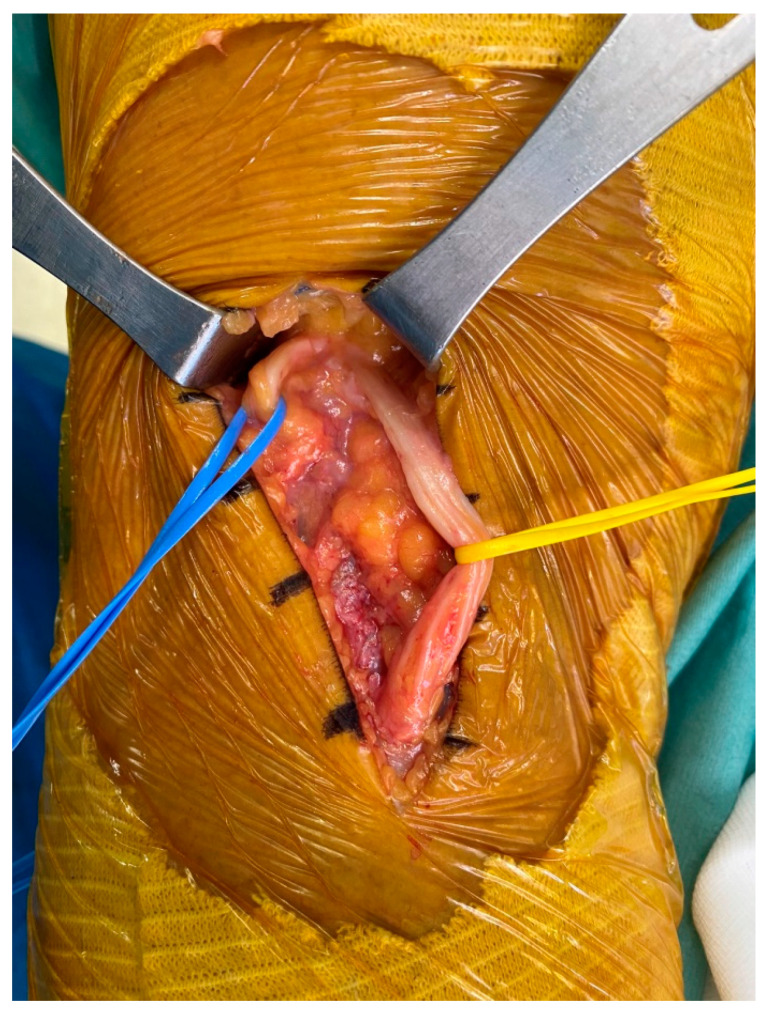
Ganglion cysts were carefully removed while both the CPN and tibial nerve were retracted to the vessel roof (blue vessel roof: CPN; yellow vessel roof: tibial nerve).

The patient was placed in the prone position under general anesthesia. A pneumatic tourniquet was applied to the left proximal thigh. After positioning the leg in the lowered position using a leg holder, the skin was prepared with betadine and routine draping was performed. The tourniquet was inflated to 300 mmHg. First, after marking the mass using ultrasound, a longitudinal skin incision was made approximately 8 cm along the tibial nerve, followed by soft tissue dissection. In the operative field, multiple lobulated ganglion cysts that originated at the site of sciatic nerve bifurcation to peroneal and tibial nerves were found. These cysts were tightly wrapped around the base of the tibial nerve and measured approximately 7 cm in size (Figure 7). When an incision was made on the wrapped sac of the tibial nerve using a No.15 blade, a ganglion pattern was observed, with jelly-like material gushing out. Both the CPN and tibial nerve were retracted using a vessel roof. The cysts were carefully dissected, taking care not to damage nerve branches such as the articular branch (Figure 8). After excision, a biopsy was requested. After confirming that there were no further compressed structures along the nerve pathway, the wound was thoroughly irrigated and repaired layer by layer. Aseptic compressive dressing and the application of a cylinder splint were also performed. The operation was then finished.

### 2.3. Postoperative Care

It was confirmed as ganglion cyst in the biopsy performed at the time of surgery. The patient did not complain of pain before or after surgery. A splint was not applied because it was judged that no benefit could be gained from immobilization. Immediately after surgery, a passive ROM exercise was performed for about 3 days. An active ROM exercise was then performed. Walker ambulation to weight-bearing ambulation were performed sequentially within two weeks.

The motor function was confirmed to be normal at 2 weeks after surgery. Sensory function was confirmed to be normal at an outpatient follow-up at 3 months. At the outpatient follow-up 1 year after surgery, an ultrasound confirmed no recurrence of cysts. Due to the patient’s dynamically improving symptoms and reluctance to undergo the EMG test due to the discomfort and pain associated with needle insertion, the EMG test was not conducted post-surgery.

## 3. Discussion

The sciatic nerve, although commonly referred to as a single entity, is typically distinctly differentiated into two separate nerves, namely the CPN and tibial nerve, as it exits beneath (or through) the piriformis muscle and extends distally [18]. The CPN originates from the lumbar and sacral regions. It starts from the dorsal branches of the L4, L5, S1, and S2 nerves. The nerve descends obliquely along the lateral side of the popliteal fossa, while approaching the medial margin of the biceps femoris muscle. Finally, it reaches the head of the fibula. The CPN wraps around the head of the fibula and branches into the deep peroneal nerve (DPN) and the superficial peroneal nerve (SPN). The SPN branches are nerves that control the peroneus longus and peroneus brevis muscles. The DPN controls muscles of the anterior compartment of the lower extremity (e.g., tibialis anterior, extensor hallucis longus, extensor digitorum longus, and peroneus tertius). These muscles are involved in the function of ankle dorsiflexion and toe extension. Thus, if the DPN becomes injured for any reason, ankle dorsiflexion and toe extension might also be affected. The DPN also controls intrinsic muscles of the foot, such as the extensor digitorum brevis muscle and the extensor hallucis brevis muscle [19,20,21]. The CPN faces the greatest risk of compression when it enters the fibular tunnel, where it runs along the lateral surface of the fibula [22]. The tibial nerve supplies muscles of the superficial and deep posterior compartments of the lower leg, including gastrocnemius, soleus, popliteus, tibialis posterior, flexor digitorum longus, and flexor hallucis longus. Considering the functions of nerves, motor weakness of ankle dorsiflexion and toe extension can be caused by peroneal nerve palsy, and motor weakness of ankle plantarflexion and toe flexion can be caused by tibial nerve palsy. As a result, the differential diagnosis of a patient experiencing unilateral inability to dorsiflex or plantarflex the ankle and extend or flex the toes, not only lesions in the L4 and L5 nerve roots, lumbosacral plexus, and sciatic nerve, but also the simultaneous entrapment of the common peroneal nerve and the tibial nerve, should be considered [18].

When peroneal nerve lesions occur at the knee or distal thigh level, patients often complain of ambulatory dysfunction, which is caused by weakness or paralysis of ankle dorsiflexors. Because the affected foot needs more lifting from the ground during the swing phase of walking in order to clear the foot, a steppage gait pattern is common [11]. By summarizing the contents of several papers, we can identify causes of peroneal nerve palsies. The list includes injuries caused by squatting or leg crossing, chronic low-grade infection, varicose veins, rapid and significant weight loss, nerve herniation through a fascial defect, prolonged pneumatic compression, and knee surgery (e.g., total knee arthroplasty and proximal tibial osteotomy) and mass (e.g., giant plexiform neurofibromatosis, schwannoma, and ganglion cyst) [22]. In patients with the aforementioned risk factors, individuals who exhibit motor weakness in ankle dorsiflexion or toe extension may be suspected to have peroneal palsy. If physicians are aware of the possibility of peroneal palsy, it is necessary to first exclude spinal diseases. Physical examination includes checking motor and sensory grades and performing a Tinel test. The Tinel test can be used to assess the presence of a tingling or prickling sensation when the injured nerve trunk is percussed at or distal to the site of the lesion [23].

In the presence of neuropathy, EMG is a useful test not only before surgery, but also during follow-up examination after surgery. Due to a considerable diversity in postsurgical neuropraxia, even with similar injury/repair patterns, patients experiencing neuropathic symptoms should be closely observed during the initial weeks following surgery [24]. Around six weeks post-operatively, an EMG can provide additional insights into the underlying cause and establish a baseline for future assessments if the symptoms persist. At this juncture, surgeons may opt for surgical intervention or choose to continue monitoring with a potential follow-up EMG at 12 weeks. However, it may not always be completely accurate. Therefore, in patients with neuropathy, it is necessary not to blindly trust the EMG result but to screen the area below or above the suspected lesion using an USG. Because an USG is fast, non-invasive, simple, and cost-effective, it can reduce risks [25]. Even though an MRI may be considered a more accurate test than an USG for detecting lesions in neuropathy, performing MRI scans for every area suspected of neuropathy is not efficient and is also costly. As a result, if screening is successful using an USG, the location and cause of the lesion can be confirmed, and a suitable treatment method can be established through additional examinations, such as an MRI.

As in the present case, there are several treatment methods for peroneal palsy caused by a ganglion. When conservatively treated, it takes 1 to 2 years to fully recover. The recovery can be incomplete. In some cases, the patient may need to use a brace for an extended period of time [26]. Some articles have introduced cases successfully treated with a simple cyst aspiration or cyst aspiration [16,17]. These methods are not favorable due to a significant risk of nerve damage, a high recurrence rate, and a low patient satisfaction. The current treatment of choice for the compression of a peroneal nerve palsy caused by a ganglion cyst is the surgical removal of the ganglion [11]. To prevent the risk of recurrence, it is important to excise the stalk and its base in the superior tibiofibular joint. Sometimes, the small sensory articular branch of the joint has to be sacrificed [27]. Recovery is much faster after operative decompression and will take place within a few days or weeks [10]. If surgery is delayed, there is a risk that the nerve damage will not be permanently repaired. Also, it is considered that the faster the operation, the better the prognosis. Thus, once diagnosed, it is recommended to undergo surgery as soon as possible without delay. If a patient with compressive neuropathy due to cyst formation exhibits minimal and nonprogressive symptoms, decompression should be contemplated within three months of symptom onset. However, if the symptoms rapidly worsen and/or the patient experiences considerable functional deficits, acute decompression may be warranted. Given the evident motor weakness and sensory deficit in this case, surgical decompression was promptly performed upon diagnosis [24].

After surgery, the patient was allowed to bear full weight, with the suggestion of using crutches in the case of severe pain. Additionally, education on physical therapy focusing on strengthening dorsiflexors and evertors was provided to address the muscle weakness caused by compressive neuropathy. In cases of severe muscle weakness, electrical stimulation may assist in contracting weakened muscles. Orthotic interventions, such as lateral wedge shoes, could also be beneficial [28,29]. For about 2 years post-surgery, neurological examinations every 3–6 months are needed to monitor recovery. Postoperative electrodiagnostic studies may aid in documenting recovery. Imaging like MRI or ultrasound should confirm the absence of cyst recurrence [29].

A limitation of this paper was that it focused on a single case, making it impossible to establish a consensus on the precise timing of surgical treatment. We could determine the usefulness of screening with USG instead of performing expensive and advanced tests such as MRI when a patient presents with motor weakness or sensory loss in the lower extremity.

## 4. Conclusions

When doctors encounter patients with neuropathy, they typically begin with physical examinations. To deduce which nerve is affected and at which level it is being compressed, it is crucial to conduct a thorough neurological assessment of the patient’s motor and sensory functions. Based on the neurological examination findings, a screening USG for suspected problematic areas can be prioritized before proceeding with a further evaluation, such as an MRI. This can reduce the unnecessary waste of resources and time consumption. Most importantly, it can reduce the risk of misdiagnosis and provide a more accurate treatment tailored to the patient.

## Data Availability

The data presented in this study are available on request from the corresponding author due to (specify the reason for the restriction).

## Abbreviations

CPN	Common peroneal nerve
DPN	Deep peroneal nerve
SPN	Superficial peroneal nerve
EMG	Electromyography
MRI	Magnetic resonance imaging
USG	Ultrasonography
